# Protective Effects of Carvacrol against Oxidative Stress Induced by Chronic Stress in Rat's Brain, Liver, and Kidney

**DOI:** 10.1155/2016/2645237

**Published:** 2016-01-19

**Authors:** Saeed Samarghandian, Tahereh Farkhondeh, Fariborz Samini, Abasalt Borji

**Affiliations:** ^1^Department of Basic Medical Sciences, Neyshabur University of Medical Sciences, Neyshabur 14139-93186, Iran; ^2^Department of Immunogenetics, BuAli Research Institute, Mashhad University of Medical Sciences, Mashhad 9196773117, Iran; ^3^Department of Neurosurgery, Faculty of Medicine, Mashhad University of Medical Sciences, Mashhad 8564-917794, Iran

## Abstract

Restraint stress may be associated with elevated free radicals, and thus, chronic exposure to oxidative stress may cause tissue damage. Several studies have reported that carvacrol (CAR) has a protective effect against oxidative stress. The present study was designed to investigate the protective effects of CAR on restraint stress induced oxidative stress damage in the brain, liver, and kidney. For chronic restraint stress, rats were kept in the restrainers for 6 h every day, for 21 consecutive days. The animals received systemic administrations of CAR daily for 21 days. To evaluate the changes of the oxidative stress parameters following restraint stress, the levels of malondialdehyde (MDA), reduced glutathione (GSH), superoxide dismutase (SOD), glutathione peroxidase (GPx), glutathione reductase (GR), and catalase (CAT) activities were measured in the brain, liver, and kidney. In the stressed animals that received vehicle, the MDA level was significantly higher (*P* < 0.001) and the levels of GSH and antioxidant enzymes were significantly lower than the nonstressed animals (*P* < 0.001). CAR ameliorated the changes in the stressed animals as compared with the control group (*P* < 0.001). This study indicates that CAR can prevent restraint stress induced oxidative damage.

## 1. Introduction

Plants products and their derivatives have been considered as an origin of therapeutic elements from ancient times. Today, there is basic research motivation in essential oils and extracts from different plant sources as potential antioxidant materials [1–5]. Carvacrol (5-isopropyl-2-methyl phenol-CAR) is an ingredient of the essential oil obtained from* Origanum hirtum*, wild bergamot, pepperwort, and several other essential oils that possesses antioxidant and antimicrobial activities and a particular aroma which makes it an attractive component for certain types of foods [6]. CAR, or cymophenol, C_6_H_3_CH_3_, is a monoterpenoid phenol. It has a characteristic pungent, warm odor of oregano. The physicochemical characteristics of CAR and its chemical structure are presented in Table 1. CAR is considered safe for consumption and as a natural replacement of synthetic antioxidative food additives [6]. Several studies have shown that CAR has antioxidant, anti-inflammatory, antitumor, analgesic, antihepatotoxic, antimicrobial, and insecticidal activities [7]. CAR has strong antioxidant property and may be effective in prevention and inhibition of several diseases [8].

Oxidative stress is made by an imbalance between the generations of reactive oxygen species (ROS) and detoxifying the reactive intermediates via biological system's ability [2, 3, 9]. Oxidative stress is a substantial mechanism that may be involved in the cytotoxicity induced by chronic stress [10]. Stress induced sympathetic stimulation causes an elevated respiration rate to generate more available oxygen for tissues. The increased metabolic rate also produces extra free radicals, leading to an imbalance between ROS generation and antioxidant system [10]. These free radical species result in oxidative damage to different molecules in cells, such as proteins, lipids, and nucleic acids [10].

It has been indicated that supplementation with natural antioxidants increases performance of the body organ during exposure to stressful environments [4, 11, 12]. CAR has strong antioxidant activity [11]. CAR treatment significantly enhances the glutathione (GSH) level whereby the maintenance of GSH by CAR occurs basically due to removal of ROS through its radical scavenging effects [11]. It has been also illustrated that CAR raises total antioxidant capacity levels in cell cultures and animals [13]. Other investigations have identified that CAR protects against different pharmacological aspects, including anxiolytic-like, antitumor, antidepressant, antinociceptive, hypotensive, and antidiabetic activities [14, 15]. Since CAR is showed to have protective effect against the function of free radicals, we hypothesized that the administration of CAR might prevent chronic stress induced tissue damage through protection against oxidative stress.

Strong evidences have indicated the effect of various antioxidants on the chronic restraint or immobilization-induced stress model [16]. Therefore, the present study was designed to investigate the effect of CAR on oxidative stress-related changes in the brain, liver, and kidney of immobilization stress.

## 2. Materials and Methods

### 2.1. Reagents

All purified enzymes, coenzymes, substrates, standards, buffers, kits, and also carvacrol and other chemicals were purchased from Sigma-Aldrich Chemical (St. Louis, USA) and corticosterone ELIZA kit was purchased from Cusabio (Cusabio Biotech Co., Ltd.).

### 2.2. Animals

Wistar albino rats (230 ± 14.5 g) were bred at the University Experimental Animal Care Centre. Animals were maintained under standard environmental conditions and had free access to standard rodent feed and water.

### 2.3. Study Design

Rats were randomly divided into eight experimental groups (8 rats per group) as follows: (1) vehicle (Veh) + no-stress (NS) (Veh-NS); (2) vehicle + stress (Veh-S), (3) CAR (20 mg/kg, IP) + no-stress (CAR20-NS), (4) CAR (30 mg/kg, IP) + no-stress (CAR30-NS); (5) CAR (40 mg/kg, IP) + no-stress (CAR40-NS), (6) CAR (20 mg/kg, IP) + stress (CAR20-S), (7) CAR (30 mg/kg, IP) + stress (CAR30-S), and (8) CAR (40 mg/kg, IP) + stress (CAR40-S). Restraint stress was performed using a rodent restrainer made of Plexiglas that closely fit to the rats' body. For chronic restraint stress, rats were kept in the restrainers for 6 h per day for 21 consecutive days. The animals received systemic administrations of vehicle (dimethyl sulfoxide, DMSO) or CAR daily for 21 days [16]. At the end of the experimental period, animals were anesthetized by ether and blood was subsequently collected from the retroorbital sinus. Blood and sera were separated by centrifugation at 5000 rpm for 5 min for corticosterone measurement. Then, brain, liver, and kidney were removed for measuring the oxidative stress markers. After the removal of tissues, they were washed in cold 0.9% saline and kept at −70°C until they were used for preparation of homogenates with a homogenizer. Each tissue was finely minced and homogenized in 50 mM phosphate buffer, pH 7.4, and centrifuged at 10,000 ×g for 15 min at 4°C (Beckman Refrigerated Ultracentrifuge). The homogenate and supernatant were used for the assays.

### 2.4. Corticosterone Evaluation

Under deep anesthesia, blood was collected from the retroorbital sinus of rats. Blood was allowed to clot and sera were separated using centrifugation at 5000 rpm for 5 min and stored at −80°C until use. Total serum level of corticosterone was measured by ELISA kits (CORT ELISA Kit CSB-E07014r).

### 2.5. Measurement of Lipid Peroxidation

Malondialdehyde (MDA) results from degradation of polyunsaturated lipids. The production of this substance is used as a biomarker to measure the level of lipid peroxidation. MDA reacts with thiobarbituric acid (TBA) as a thiobarbituric acid reactive substances (TBARS) to form a 1 : 2 MDA-TBA adduct, which is absorbed at 532 nm. Thus, the quantity of TBARS is proportionate to the amount of MDA. Concentration of TBARS is determined according to a method of Uchiyama and Mihara. The concentration of TBARS was calculated using MDA standard curve and was expressed as nmol/mg of protein [17].

### 2.6. Estimation of GSH

GSH was measured by the method of Beutler et al. [18]. Briefly, to 0.1 mL of sample, 0.9 mL distilled water and 1.5 mL of precipitating reagent were added (3.34 g metaphosphoric acid, 0.4 g EDTA, and 60.0 g sodium chloride). Tubes were shaken and allowed to stand for 5 min at room temperature (25 ± 1°C). The mixture was centrifuged for 15 min at 4000 rpm at 4°C. In 1.0 mL supernatant, 4.0 mL of phosphate solution (0.3 M disodium hydrogen phosphate) and 0.5 mL 5-50-dithiobis-(2-nitrobenzoic acid) (DTNB) (80 mg in 1% sodium citrate) were added. The development of yellow color complex was read immediately at 412 nm on a spectrophotometer. A standard curve using GSH was prepared and GSH concentration in the experimental samples was extrapolated from the standard curve. GSH concentration was calculated and expressed as *μ*mol of GSH/mg protein.

### 2.7. Measurements of Enzymes

The activity of SOD was determined by the method of S. Marklund and G. Marklund [19], using inhibition of pyrogallol autoxidation at pH 8. The specific activity of SOD is expressed as units per mg protein per minute. The activity of GPx was measured by the method of Paglia and Valentine [20]. GPx catalyses the oxidation of glutathione by cumene hydroperoxide. In the presence of glutathione reductase (GR) and NADPH the oxidized glutathione is immediately converted to the reduced form with a concomitant oxidation of NADPH to NADP. The decrease in absorbance at 340 nm is measured. GR catalyses the reduction of glutathione in the presence of NADPH, which is oxidized to NADP. The decrease in absorbance at 340 nm is measured. The levels of GPx and GR were expressed as U/mg protein. CAT activity was assayed by H_2_O_2_ consumption, following Aebi's [21] method and modified by Pieper et al. [22].

### 2.8. Protein Estimation

Protein was estimated in subcellular fractions by the method of Bradford [23] using bovine serum albumin (BSA) as standard.

### 2.9. Statistical Analysis

All experiments were carried out at least in duplicate. Each group consisted of eight rats. One-way analysis of variance (ANOVA) was performed and Tukey* post hoc* test was used for multiple comparisons. Statistical analyses were performed using the InStat 3.0 program. The results are expressed as mean ± SEM. The results originated from analysis of serum. Differences of *P* < 0.05 were considered significant.

## 3. Results

The levels of MDA, GSH, SOD, CAT, GPx, and GR in brain, liver, and kidney in the all groups are shown in Tables 2, 3, and 4. The MDA level of the Veh-S group in all tissues was significantly higher than those of Veh-NS and three CAR-NS groups (*P* < 0.001). The MDA level in the CAR40-S group in all tissues was significantly lower than those of the Veh-S group (*P* < 0.001). Our data showed that there was a significant difference in the MDA level in CAR40-S and CAR20-S groups in all tissues (*P* < 0.001). The MDA level of the CAR30-S in the brain and liver was significantly lower than CAR20-S group (*P* < 0.01). In addition, the MDA level of the CAR40-S in the brain was significantly lower than the CAR30-S group (*P* < 0.01).

The GSH level of the Veh-S group in all tissues was significantly lower than those of Veh-NS and three CAR-NS groups (*P* < 0.001 for brain and kidney; *P* < 0.01 for liver). The GSH level in the CAR40-S group in brain, liver, and kidney tissues was significantly higher than those of the Veh-S group (*P* < 0.001, *P* < 0.05, and *P* < 0.001, resp.). Our results illustrated that there was a significant difference between GSH level in the CAR40-S and the CAR20-S groups in the brain and kidney (*P* < 0.001). In addition, the GSH level of the CAR40-S in the brain was significantly higher than CAR30-S group (*P* < 0.01).

The activities of SOD (*P* < 0.001 for brain and kidney; *P* < 0.05 for liver), GPx (*P* < 0.01 for brain and kidney; *P* < 0.001 for liver), GR (*P* < 0.001 for all tissues), and CAT (*P* < 0.001 for brain and kidney; *P* < 0.01 for liver) in the Veh-S group in all tissues were significantly lower than those of Veh-NS and three CAR-NS groups. The SOD (*P* < 0.001 for brain; *P* < 0.05 for liver; *P* < 0.01 for kidney), GPx (*P* < 0.05 for all tissues), GR (*P* < 0.05 for all tissues), and CAT (*P* < 0.001 for brain; *P* < 0.05 for liver; *P* < 0.01 for kidney) activities in the CAR40-S group in all tissues were significantly higher than those of the Veh-S group. In addition, the SOD and CAT activities in the CAR30-S group in brain were significantly higher than those of the Veh-S group (*P* < 0.01). Present data indicated that there was a significant difference between the SOD (*P* < 0.001 for brain, *P* < 0.05 for kidney), GPx (*P* < 0.05 for kidney), and CAT (*P* < 0.001 for brain, *P* < 0.01 for kidney) activities in the CAR40-S and the CAR20-S groups in the brain and kidney. The significant difference was also observed in the CAT activity in the CAR40-S and the CAR30-S groups in the kidney (*P* < 0.05).

**Figure 1:**
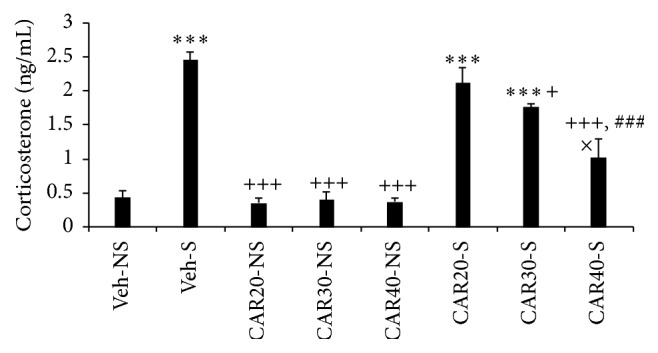
Effect of CAR on corticosterone levels in serum of immobilization stress and control groups (*n* = 8, for each group). Each measurement was done at least in triplicate and the values are the means ± SEM for eight rats in each group. Significantly different from Veh-NS groups (^*∗∗∗*^ *P* < 0.001). Significantly different from Veh-S groups (^+^ *P* < 0.05, ^+++^ *P* < 0.001). Significant difference in CAR20-S versus CAR30-S and CAR40-S groups (^###^ *P* < 0.001). Significant difference in CAR30-S group versus CAR40-S group (^×^ *P* < 0.05).

The serum corticosterone level of the Veh-S group was significantly higher than those of Veh-NS and three CAR-NS groups (*P* < 0.001). The serum corticosterone level in the CAR30-S and CAR40-S groups was significantly lower than those of the Veh-S group (*P* < 0.05, *P* < 0.001, resp.). The significant difference was observed in the serum corticosterone level in the CAR40-S and the CAR20-S and CAR30-S groups (*P* < 0.001, *P* < 0.05, resp.) (Figure 1).

## 4. Discussion

The present study indicates that chronic restraint stress induces oxidative stress in the brain, liver, and kidney and this oxidative stress damage in the tissues ameliorated by CAR treatment. Present data shows that CAR is effective against oxidative damage induced by chronic stress in the main organs. In this investigation MDA, marker for lipid per oxidation, exhibits the oxidative damage and the reduction of antioxidants. The levels of antioxidants including GSH, SOD, GPx, GR, and CAT were evaluated in the brain, liver, and kidney of rats exposed to restraint stress to determine the antioxidative potential of CAR. In the untreated control animals exposed to restraint stress, there was a considerable increase in the brain, liver, and kidney MDA levels, proposing stress induced lipid peroxidation. Present data are in agreement with the previous observations of increased levels of lipid peroxides in the brain, liver, and kidney of rats exposed to restraint stress [24]. The increase in tissue MDA level in the untreated control animals exposed to restraint stress accompanied by significant decrease in the GSH, SOD, GPx, GR, and CAT levels showed the overproduction of free radicals during immobilization stress. Treatment of animals with CAR resulted in decrease in the tissue MDA level and increase in the GSH, SOD, GPx, GR, and CAT activities levels, in comparison to the untreated exposed animals. The GSH level exhibits an essential role in detoxification in the tissue [25–28]. In the present study, GSH level was decreased in tissues of stress groups [29]. Stress decreases the GSH level and leads to increased levels of ROS in rat tissues [28]. Strong evidence has been indicated that the enzymatic antioxidant defense system against hydrogen peroxide (H_2_O_2_), which is the high toxic substance for tissues, is primarily mediated by the GSH system [4, 25]. GSH, as a cofactor of glutathione peroxidase (GPx), exhibits an essential role in the cell defense system. GSH, a thiol compound, has antioxidant activity in cells and reduces H_2_O_2_ and organic peroxides formation during lipid peroxidation with formation of oxidized glutathione disulfide (GSSG) [30]. In a normal physiological situation, glutathione exists as the reduced form (GSH) in cell; however, GSH is changed into its oxidized form (GSSG) by glutathione reductase (GR) when cells are exposed to overproduction of free radicals [2, 3, 31]. The peroxidase/glutathione reductase redox cycle is responsible for the maintenance of proper GSH concentration [32]. Changes in the activity of GPx and GR can disturb the concentration of GSH level [32]. In this study, treatment with CAR ameliorated the oxidative stress induced decrease in the GSH, SOD, GPx, GR, and CAT levels. SOD is a main antioxidant enzyme for scavenging superoxide anion. The activity of SOD which decreased in rat liver, brain, and kidney [29, 33] during restraint stress constitutes an important defense system to clear up ROS* in vivo*.

Our results confirmed the previous studies so that restraint stress induces free radical production and decreases antioxidant enzyme activities. In the physiological aspects, stress induces oxygen free radicals generation mostly formed in mitochondria, peroxisomes, lysosomes, cytosol, and the plasma membrane in body [34]. However, in the biochemical view, an imbalance between ROS generation and its clearance by the antioxidant defense system in the body has been observed in brain, liver, and kidney damage [28, 35]. The produced free radicals from cellular metabolic processes have long been involved in the cellular toxicity [35, 36]. An immobilization stress response results in the overproduction of free radicals that leads to lipid peroxidation, particularly in cell membranes [37]. The lipid peroxidation can change membrane integrity and then leads to tissue damage [10, 36]. Pervious findings demonstrated that antioxidant enzymes activity was lower in liver, brain, and kidney after immobilization stress in animals [38]. In addition, the MDA content increased in all tissues especially in brain [38]. Similarly, present study indicated that oxidative stress induces oxidative injury in brain, liver, and kidney via increasing the MDA level and decreasing the GSH and antioxidant enzymes activity. We also observed that CAR ameliorated these modifications.

In this study, serum corticosterone level was measured in the rats immediately after chronic stress. According to the present findings, chronic stress increased serum corticosterone and CAR significantly decreased corticosterone level. Glucocorticoids exert an essential role in chronic stress induced oxidative injury [39]. Glucocorticoids may enhance the tissue MDA in stressed rats and this is a direct relation between serum corticosterone and the liver MDA level [39, 40]. Furthermore, raised levels of glucocorticoids during restraint stress may affect the animal antioxidant content [39]. The mechanisms illustrated above elaborated in tissue damage induced by oxidative stress in current study.

The present investigation indicates that the injurious effects of chronic stress ameliorated by CAR treatment, proposing a protective effect of these agents against chronic stress. CAR also prevents lipid peroxidation by inducing SOD, GPx, GR, and CAT. CAR efficiently scavenges free radicals such as peroxyl radicals, superoxide radicals, hydrogen peroxide, and nitric oxide [41, 42]. CAR exerts antioxidant effect both* in vitro* and* in vivo* and its antioxidant activity is attributed to the presence of hydroxyl group (OH^∙^) linked to aromatic ring [43, 44]. The increased levels of lipid peroxidation products in plasma, liver, kidney, and liver and the decreased levels of enzymic and nonenzymic antioxidants in rat were restored to normalcy after CAR treatment. CAR treatment inhibited free radicals formation and lipid peroxidation levels, which further improves membrane fluidity. CAR has been found to act as a radical scavenger inhibiting lipid peroxidation* in vivo* and* in vitro*.

In conclusion, the present study shows that CAR can inhibit chronic stress induced oxidative damage of the brain, liver, and kidneys. Thus, CAR should be fruitful as new pharmacological agent for ameliorating chronic stress induced oxidative damage.

## Figures and Tables

**Table 1:** Physicochemical characteristics and molecular structure of carvacrol at 25°C.

Aroma compound	Molecular structure	Molecular weight (g mol^−1^)	Density (kg/m^3^)	Vapour pressure 25°C (pa)	Maximum solubility in water (g L^−1^)
Carvacrol	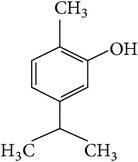	150.22	977.2	6.4	0.11

**Table 2:** Effect of CAR on MDA (nmol/mgp), GSH (*µ*mol/mgp), SOD (U/mgp), GPx (U/mgp), GR (U/mgp), and CAT (U/mgp) levels in brain of immobilization stress and control groups (*n* = 8, for each group).

Brain	MDA	GSH	SOD	GPx	GR	CAT
Veh-NS	2.68 ± 0.12	8.11 ± 0.40	3.12 ± 0.10	1.03 ± 0.15	0.85 ± 0.11	5.01 ± 0.35
Veh-S	6.28 ± 0.24^*∗∗∗*^	3.67 ± 0.15^*∗∗∗*^	0.98 ± 0.13^*∗∗∗*^	0.37 ± 0.10^*∗∗*^	0.29 ± 0.12^*∗∗*^	2.47 ± 0.11^*∗∗∗*^
CAR20-NS	2.23 ± 0.18^+++^	8.01 ± 0.39^+++^	3.06 ± 0.16^+++^	0.96 ± 0.13^+^	0.93 ± 0.08^+++^	4.87 ± 0.18^+++^
CAR30-NS	2.41 ± 0.21^+++^	7.89 ± 0.45^+++^	2.92 ± 0.20^+++^	1.11 ± 0.12^+++^	0.87 ± 0.13^++^	5.10 ± 0.15^+++^
CAR40-NS	2.36 ± 0.17^+++^	7.95 ± 0.48^+++^	3.23 ± 0.15^+++^	1.17 ± 0.10^+++^	0.90 ± 0.10^++^	5.07 ± 0.21^+++^
CAR20-S	5.13 ± 0.25^*∗∗∗*++^	4.02 ± 0.22^*∗∗∗*^	1.35 ± 0.28^*∗∗∗*^	0.55 ± 0.05	0.43 ± 0.09	2.93 ± 0.20^*∗∗∗*^
CAR30-S	3.98 ± 0.11^*∗∗∗*+++##^	5.18 ± 0.29^*∗∗∗*^	2.07 ± 0.19^*∗∗*++^	0.74 ± 0.09	0.52 ± 0.10	3.72 ± 0.13^++*∗∗*^
CAR40-S	2.87 ± 0.19^+++###××^	7.26 ± 0.30^+++###××^	2.85 ± 0.24^+++###^	0.96 ± 0.14^+^	0.74 ± 0.05^+^	4.57 ± 0.26^+++###^

Each measurement was done at least in triplicate and the values are the means ± SEM for eight rats in each group.

Significantly different from Veh-NS groups (^*∗∗*^ *P* < 0.01, ^*∗∗∗*^ *P* < 0.001).

Significantly different from Veh-S groups (^+^ *P* < 0.05, ^++^ *P* < 0.05, and ^+++^ *P* < 0.001).

Significant difference in CAR20-S versus CAR30-S and CAR40-S groups (^##^ *P* < 0.01, ^###^ *P* < 0.001).

Significant difference in CAR30-S group versus CAR40-S group (^××^ *P* < 0.01).

**Table 3:** Effect of CAR on MDA (nmol/mgp), GSH (*µ*mol/mgp), SOD (U/mgp), GPx (U/mgp), GR (U/mgp), and CAT (U/mgp) levels in liver of immobilization stress and control groups (*n* = 8, for each group).

Liver	MDA	GSH	SOD	GPx	GR	CAT
Veh-NS	1.14 ± 0.25	10.09 ± 1.08	5.39 ± 0.65	1.04 ± 0.09	0.67 ± 0.11	6.09 ± 0.24
Veh-S	3.97 ± 0.16^*∗∗∗*^	4.56 ± 0.76^*∗∗*^	2.73 ± 0.39^*∗*^	0.45 ± 0.10^*∗∗∗*^	0.24 ± 0.09^*∗∗*^	2.98 ± 0.44^*∗∗*^
CAR20-NS	0.98 ± 0.10^+++^	9.81 ± 0.90^++^	5.09 ± 0.78^+^	1.01 ± 0.02^++^	0.71 ± 0.03^++^	5.87 ± 0.76^++^
CAR30-NS	1.06 ± 0.19^+++^	10.16 ± 1.06^++^	5.12 ± 0.44^+^	0.89 ± 0.06^+^	0.60 ± 0.05^+^	5.67 ± 0.81^+^
CAR40-NS	1.20 ± 0.21^+++^	10.00 ± 0.95^++^	7.23 ± 0.27^+^	1.12 ± 0.14^+++^	0.58 ± 0.07^++^	6.12 ± 0.51^++^
CAR20-S	3.01 ± 0.11^*∗∗∗*++^	6.12 ± 1.11	3.01 ± 0.32^*∗*^	0.58 ± 0.03^*∗*^	0.42 ± 0.01	3.72 ± 0.38
CAR30-S	2.20 ± 0.14^*∗∗∗*+++##^	7.46 ± 0.50	4.12 ± 0.50	0.69 ± 0.07	0.52 ± 0.13	4.53 ± 0.53
CAR40-S	1.58 ± 0.15^+++###^	8.96 ± 1.09^+^	5.06 ± 0.21^+^	0.91 ± 0.13^+^	0.61 ± 0.08^+^	5.71 ± 0.32^+^

Each measurement was done at least in triplicate and the values are the means ± SEM for eight rats in each group.

Significantly different from Veh-NS groups (^*∗*^ *P* < 0.05, ^*∗∗*^ *P* < 0.01, and ^*∗∗∗*^ *P* < 0.001).

Significantly different from Veh-S groups (^+^ *P* < 0.05, ^++^ *P* < 0.05, and ^+++^ *P* < 0.001).

Significant difference in CAR20-S group versus CAR20-S group (^##^ *P* < 0.01, ^###^ *P* < 0.001).

**Table 4:** Effect of CAR on MDA (nmol/mgp), GSH (*µ*mol/mgp), SOD (U/mgp), GPx (U/mgp), GR (U/mgp), and CAT (U/mgp) levels in kidney of immobilization stress and control groups (*n* = 8, for each group).

Kidney	MDA	GSH	SOD	GPx	GR	CAT
Veh-NS	1.79 ± 0.33	7.65 ± 0.36	4.12 ± 0.27	2.34 ± 0.15	1.88 ± 0.07	5.01 ± 0.24
Veh-S	4.08 ± 0.29^*∗∗∗*^	3.81 ± 0.41^*∗∗∗*^	2.51 ± 0.11^*∗∗∗*^	0.86 ± 0.12^*∗∗*^	1.02 ± 0.11^*∗∗*^	2.73 ± 0.11^*∗∗∗*^
CAR20-NS	1.67 ± 0.21^+++^	7.29 ± 0.28^+++^	4.01 ± 0.14^++^	2.05 ± 0.10	1.68 ± 0.23^+^	4.89 ± 0.31^+++^
CAR30-NS	1.83 ± 0.38^+++^	7.83 ± 0.47^+++^	3.87 ± 0.33^++^	2.11 ± 0.23	1.93 ± 0.14^+++^	5.22 ± 0.42^+++^
CAR40-NS	1.74 ± 0.41^+++^	7.08 ± 0.32^+++^	4.28 ± 0.10^+++^	1.92 ± 0.27^+^	2.03 ± 0.10^+++^	5.10 ± 0.23^+++^
CAR20-S	3.77 ± 0.25	4.65 ± 0.12^*∗∗∗*^	2.88 ± 0.20	1.02 ± 0.48	1.24 ± 0.05	2.65 ± 0.27^*∗∗∗*^
CAR30-S	2.90 ± 0.17	5.85 ± 0.22^*∗∗*++^	3.43 ± 0.45	1.37 ± 0.26	1.36 ± 0.17	3.12 ± 0.51^*∗∗*^
CAR40-S	1.93 ± 0.46^+++##^	6.88 ± 0.31^+++###^	4.05 ± 0.19^++#^	2.25 ± 0.33^+#^	1.72 ± 0.16^+^	4.55 ± 0.18^++##×^

Each measurement was done at least in triplicate and the values are the means ± SEM for eight rats in each group.

Significantly different from Veh-NS groups (^*∗∗*^ *P* < 0.01, ^*∗∗∗*^ *P* < 0.001).

Significantly different from Veh-S groups (^+^ *P* < 0.05, ^++^ *P* < 0.05, and ^+++^ *P* < 0.001).

Significant difference in CAR20-S versus CAR30-S and CAR40-S groups (^#^ *P* < 0.05, ^##^ *P* < 0.01, and ^###^ *P* < 0.001).

Significant difference in CAR30-S group versus CAR40-S group (^×^ *P* < 0.05).
