# Correction to “Protective Effects of Carvacrol against Oxidative Stress Induced by Chronic Stress in Rat’s Brain, Liver, and Kidney”

**DOI:** 10.1155/bri/9836253

**Published:** 2026-11-05

S. Samarghandian, T. Farkhondeh, F. Samini, and A. Borji, “Protective Effects of Carvacrol against Oxidative Stress Induced by Chronic Stress in Rat’s Brain, Liver, and Kidney,” *Biochemistry Research International*, 2016, 2645237, https://doi.org/10.1155/2016/2645237.

In the article, there are minor typographical errors:

• In Table 3, “Significant difference in CAR20‐S group versus CAR20‐S group” should read “Significant difference in CAR20‐S group versus CAR30‐S and CAR40‐S groups.”
• In the Discussion, “pervious findings” should read “previous findings”
• In the Introduction, the incomplete chemical formula was stated for cymophenol. The correct formula is (CH_3_)_2_CHC_6_H_3_(CH_3_)OH.

We apologize for these errors.

